# Expression of miR-4739 in Gastric cancer and its Relationship with Clinical Pathological Features of Patients

**DOI:** 10.3389/fsurg.2022.897583

**Published:** 2022-05-03

**Authors:** Jiaxing Wei, Jun Li, Dong Geng, Yiling Peng, Bin Yang, Huixian Wu, Yun Zhou

**Affiliations:** National Demonstration Center for Experimental General Medicine Education, Xianning Medical College, Hubei University of Science and Technology, Xianning, Hubei, China

**Keywords:** gastric cancer, miR-4739, diagnostic value, clinical features, prognosis, influencing factors

## Abstract

**Objective:**

To investigate the expression level of miR-4739 in gastric cancer (GC), analyze its diagnostic value in GC and the relationship with clinical pathological characteristics, and analyze its impact on the prognosis of patients.

**Methods:**

A total of 96 patients with GC who underwent radical gastrectomy in our hospital from March 2017 to June 2021 were selected. GC tissues from all patients were collected, and normal tissues adjacent to cancer were collected as controls. The expression level of miR-4739 in tissues was detected, the relationship between miR-4739 and different pathological features was analyzed, and the diagnostic value of miR-4739 in GC was analyzed. All patients were followed up after the operation, and the survival time of the patients was set as from the day of the first operation to 1 d when the patients died or the follow-up ended.

**Results:**

The relative expression level of miR-4739 in the GC tissue was (0.39 ± 0.06), lower than that in the paracancerous tissue (1.18 ± 0.19) (*P* < 0.05). The AUC of miR-4739 in the diagnosis of GC was 0.705. When the Youden index was 0.320 and the optimal cutoff value was 0.37, the sensitivity was 95.30% and the specificity was 36.70%. The expression level of miR-4739 in our patient was related to the differentiation degree, lymph node metastasis, tumor diameter, and TNM stage (*P* < 0.05). During the follow-up period, 26 of 96 patients died, and the survival rate was 72.92% (26/96). The median survival time was 29 months in the miR-4739 LE group, which was shorter than 39 months in the miR-4739 HE group (*P* < 0.05). Univariate analysis showed that age, degree of differentiation, lymph node metastasis, tumor diameter, TNM staging, and miR-4739 expression were all related to the prognosis of the patient (*P* < 0.05). Multivariate analysis showed that differentiation degree, lymph node metastasis, tumor diameter, TNM staging, and miR-4739 expression were all independent factors affecting the prognosis of the patients (*P* < 0.05).

**Conclusion:**

The expression of miR-4739 in GC tissue was down-regulated, and its level was related to the degree of differentiation, lymph node metastasis, tumor diameter, and TNM stage. The expression level of miR-4739 has certain diagnostic value for patients with GC, and the prognosis of patients in LE group was worse than that in HE group.

## Introduction

Gastric cancer (GC) is a common gastrointestinal malignant tumor in clinic. In China, the incidence rate of GC in gastrointestinal malignant tumors ranks first, which seriously threatens the life quality of patients (1, 2). For the past few years, with the development of medical technology, the treatment of GC has made some progress, but the overall therapeutic effect and prognosis of patients with GC are still poor (3, 4). The occurrence of GC may be related to factors such as geographical, environmental, living habits, helicobacter pylori infection, precancerous lesions, heredity and genes, etc. Patients usually have no specific clinical manifestation at the initial stage of onset. Some patients have such symptoms as nausea and vomiting, and the diagnosis of early GC is difficult (5, 6). Up to now, the specific pathogenesis of GC is still unclear. At present, among patients diagnosed with GC in hospitals, about 90% of patients with advanced GC accompanied by different degrees of lymph node metastasis. The study showed that the early stage of GC treatment had a great influence on the therapeutic effect. The inpatients with GC had a late stage of disease, and the 5-year survival rate after radical gastrectomy in large general hospitals was about 35%. Among them, the average 5-year survival rate of patients with early GC is more than 90%, while the average 5-year survival rate of patients with advanced GC is poor, even less than 10% (2, 7). Therefore, in order to improve the prognosis of patients, clinical efforts have been made to find markers for early GC. Micro ribonucleic acid (miRNA) is an endogenous non-coding small RNA, which has been found to be extensively involved in the proliferation and migration of tumor cells (8, 9). The preliminary studies on miRNA mainly focus on the target genes and expression profiles of miRNA in different tissues and different tumor tissues. GC is usually characterized by abnormal expression of multiple mirnas, so it is of great significance to find specific mirnas for the diagnosis and treatment of GC (10, 11). Abnormal expression of miRNA is related to the occurrence of many tumors, and the maladjustment of miRNA will not only affect the function of intercellular regulatory factors, but also affect tumor growth and invasion (12, 13). Wang (14) showed that miR-4739 is related to tumor progression and plays an inhibitory role in prostate cancer, and down-regulation of its expression can promote the proliferation and migration of prostate cancer cells. Studies on GC have shown that when the malignancy of tumor cells decreases, the expression level of miR-4739 is up-regulated (15). The purpose of this study was to investigate the expression level of miR-4739 in GC, analyze its diagnostic value for GC and the relationship with clinical and pathological characteristics, and analyze its impact on the prognosis of patients.

## Materials and Methods

### Patients

A total of 96 patients with GC who underwent radical gastrectomy in our hospital from March 2017 to June 2021 were selected. There were 54 males and 42 females, aged from 32 to 78 years, with an average age of (61.34 ± 8.52) years. Tumor node metastasis (TNM) stages included stage I (*n* = 36), stage II (*n* = 30), stage III (*n* = 26) and stage IV (*n* = 14). Differentiation degree: 58 cases with low differentiation, 22 cases with medium differentiation and 16 cases with high differentiation. Inclusion criteria: All met the diagnostic criteria of GC (16); All patients underwent radical gastrectomy. No radiotherapy or chemotherapy was given before admission; Patients with clear clinical stages and pathologic differentiation. Exclusion criteria: patients with immune system diseases; Patients with malignant tumors in other parts; Long-term use of hormones or non-steroidal anti-inflammatory drugs; Patients with liver and kidney dysfunction, endocrine diseases and blood system diseases; Patients with incomplete clinical data or lost follow-up process. GC tissues of all patients were collected, and normal adjacent tissues (more than 5 cm away from the tumor boundary and confirmed by pathology to have no infiltration of carcinomatous tissues) were collected as controls. All tissue samples were excised, rinsed with PBS, placed in liquid nitrogen for temporary storage, and quickly transferred to −80°C for storage after quick-freezing overnight.

### Expression of miR-4739 Detected by Real-Time Quantitative PCR (qRT-PCR)

The total RNA in GC tissues and adjacent tissues was extracted by Trizol method. Detect that molecular weight of the RNA by adopting gel electrophoresis; RNA concentration was detected by spectrophotometer. The RT reaction was performed using ONE STEP PrimeScript cDNA Synthesis Kit (Bao Biological Engineering Dalian Co., LTD) with DNA as the template. The 20 µL PCR reaction system was prepared in an ice bath. The primers were designed according to the data obtained from NCBI database. The primer sequences were as follows: upstream primer of miR-4739: 5′-GCTGGGACATTGAAAGTCTCA-3′; Downstream primer: 5′-GATTTCCCATCGGCGTC-3′. Upstream primer internal reference U6, 5′-CTCGCTTCGGCAGCCACA-3, and downstream primer, 5′-AAGCTTCACGAATTTGCGT-3′. The baseline was adjusted according to the instructions, the threshold was set in the linear part of the log plot of fluorescence values, and the Ct values of the miR-4739 band and the internal reference U6 band were read from the software. Δct = mean sample ct−mean internal reference ct, ΔΔct = Δct- (mean random negative control sample Ct−mean internal reference Ct), and 2^−ΔΔCt^ was used to represent the relative expression level of miR-4739.

### Follow-Up

Patients were followed-up by outpatient or telephone every 3 months after operation. The deadline of follow-up was December 2021. The survival time of the patient was set as 1 d from the date of the first operation to the date of her death or the end of her follow-up. According to the survival state of the patients, they were divided into the survival group and the death group.

### Statistical Methods

All data were processed with SPSS 22.0 statistical software. The enumeration data were examined by X^2^ test and expressed by [n(%)], the measurement data were examined by t-test and expressed by $\left(\bar{x}\pm s\right)$. Multivariate analysis adopts multiple Logistic regression model. The ROC curve was used to analyze the diagnostic value of miR-4739 in GC patients. Kaplan-meier survival curve was used to analyze the relationship between Mir-4739 and prognosis of gastric cancer patients, and log-rank test was used for comparison. The difference is statistically significant when *P* < 0.05.

## Results

### Expression of miR-4739 in GC Tissue and Paracancerous Tissue

**Figure 1:**
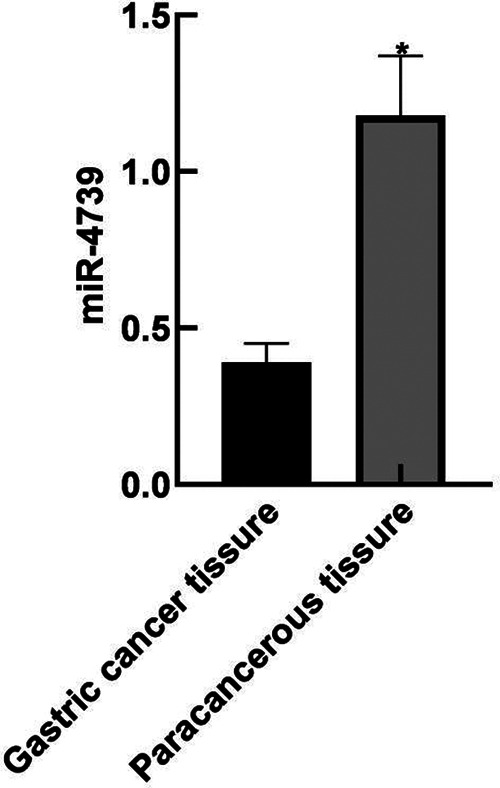
Expression of miR-4739 in GC tissue and paracancerous tissue. Note: Compared with gastric cancer tissue, *P < 0.05.

The relative expression level of miR-4739 in the GC tissue was (0.39 ± 0.06), lower than that in the paracancerous tissue (1.18 ± 0.19) (*P* < 0.05). As shown in Figure 1.

### Diagnostic Value of miR-4739 Expression Level in GC

**Figure 2:**
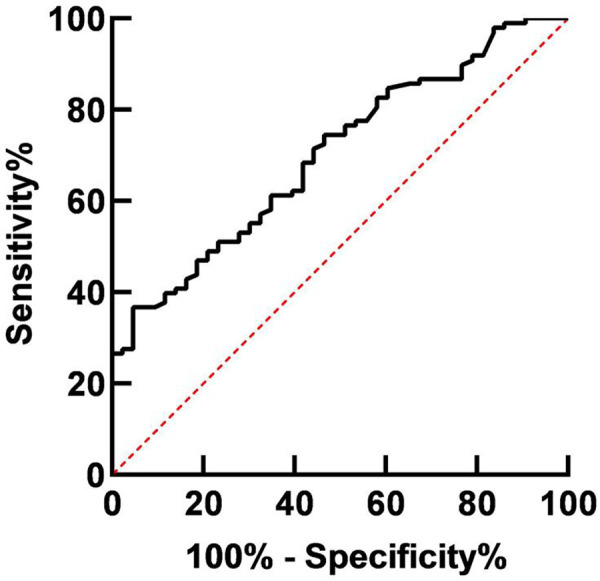
Diagnostic value of miR-4739 expression level in GC.

The AUC of miR-4739 in the diagnosis of GC was 0.705. When the Youden index was 0.320 and the optimal cutoff value was 0.37, the sensitivity was 95.30% and the specificity was 36.70%. As shown in Table 1 and Figure 2.

**Table 1:** Diagnostic value of miR-4739 expression level in GC.

Predictive indexes	AUC	95%CI	Youden index	Sensitivity (%)	Specificity (%)	Cut-off value
miR-4739	0.702	0.614–0.790	0.320	95.30%	36.70%	0.37

### Relationship between miR-4739 and Clinical Pathological Characteristics

The cells were divided into high-expression (HE) group (>0.37) and low-expression (LE) group (≤0.37) with the best cutoff value of miR-4739 as the boundary. The expression level of miR-4739 in our patient was related to the differentiation degree, lymph node metastasis, tumor diameter, and TNM stage (*P* < 0.05). As shown in Table 2.

**Table 2:** Relationship between miR-4739 expression level and clinical pathological features (n,%).

Clinical pathological features	*n*	miR-4739		χ^2^	*P*
		HE group (*n* = 55)	LE group (*n* = 41)		
Age	** **	** **	** **	1.336	0.248
≥60 years old	52	27(49.09)	25(60.98)	** **	** **
<60 years old	44	28(50.91)	16(39.02)	** **	** **
Gender	** **	** **	** **	0.649	0.421
Man	54	29(52.73)	25(60.98)	** **	** **
Woman	42	26(47.27)	16(39.02)	** **	** **
Degree of differentiation	** **	** **	** **	4.868	0.027
Low differentiation	58	28(50.91)	30(73.17)	** **	** **
Moderate to high differentiation	38	27(49.09)	11(26.83)	** **	** **
Lymph node metastasis	** **	** **	** **	5.248	0.022
Yes	60	29(52.73)	31(75.61)	** **	** **
No	36	26(47.27)	10(24.39)	** **	** **
Tumor diameter	** **	** **	** **	5.243	0.022
≥5 cm	41	18(32.73)	23(56.10)	** **	** **
<5 cm	55	37(67.27)	18(43.90)	** **	** **
TNM staging	** **	** **	** **	4.234	0.040
Stages I–II	56	37(67.27)	19(46.34)	** **	** **
Stages III–IV	40	18(32.73)	22(53.66)	** **	** **

### Relationship between miR-4739 Expression Level and Prognosis of Patients with GC

**Figure 3:**
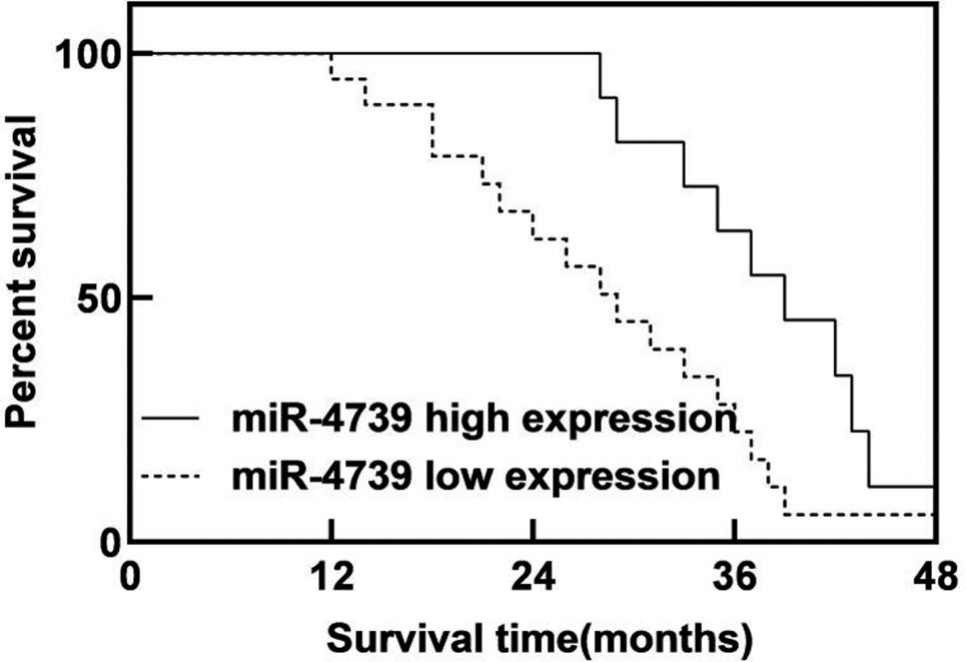
Relationship between miR-4739 expression level and prognosis of patients with GC.

During the follow-up period, 26 of 96 patients died, and the survival rate was 72.92%(26/96). The median survival time was 29 months in the miR-4739 LE group, which was shorter than 39 months in the miR-4739 HE group (*P* < 0.05). As shown in Figure 3.

### Univariate Analysis of Prognosis of Patients with GC

Univariate analysis showed that age, degree of differentiation, lymph node metastasis, tumor diameter, TNM staging, and miR-4739 expression were all related to the prognosis of the patient (*P* < 0.05). As shown in Table 3.

**Table 3:** Univariate analysis of prognosis of patients with GC (*n*,%).

Factor	n	Survival group (*n* = 70)	Death group (*n* = 26)	χ^2^	*P*
Age	** **	** **	** **	5.136	0.023
≥60 years old	52	33 (47.14)	19 (73.08)	** **	** **
<60 years old	44	37 (52.86)	7 (26.92)	** **	** **
Gender	** **	** **	** **	0.405	0.524
Man	54	38 (54.29)	16 (61.54)	** **	** **
Woman	42	32 (45.71)	10 (38.46)	** **	** **
Degree of differentiation	** **	** **	** **	6.176	0.013
Low differentiation	58	37 (52.86)	21 (80.77)	** **	** **
Moderate to high differentiation	38	33 (47.14)	5 (19.23)	** **	** **
Lymph node metastasis	** **	** **	** **	7.441	0.006
Yes	60	38 (54.29)	22 (84.62)	** **	** **
No	36	32 (45.71)	4 (15.38)	** **	** **
Tumor diameter	** **	** **	** **	5.167	0.023
≥5 cm	41	25 (35.71)	16 (61.54)	** **	** **
<5 cm	55	45 (64.29)	10 (38.46)	** **	** **
TNM staging	** **	** **	** **	8.253	0.004
Stages I–II	56	47 (67.14)	9 (34.62)	** **	** **
Stages III–IV	40	23 (32.86)	17 (65.38)	** **	** **
Expression of miR-4739	** **	** **	** **	7.494	0.006
High	55	46 (65.71)	9 (34.62)	** **	** **
Low	41	24 (34.29)	17 (65.38)	** **	** **

### Analysis of Multiple Factors Affecting the Prognosis of Patients with GC

Multivariate analysis showed that differentiation degree, lymph node metastasis, tumor diameter, TNM staging, and miR-4739 expression were all independent factors affecting the prognosis of the patients (*P* < 0.05). As shown in Table 4 and Table 5.

**Table 4:** Multi-factor analysis assignment table.

Factors	Variable	Assignment
Age	X1	<60 = 0, ≥60 = 1
Degree of differentiation	X2	Medium to high differentiation = 0, low differentiation = 1
Lymph node metastasis	X3	No = 0, yes = 1
Tumor diameter	X4	<5 cm = 0, ≥5 cm = 1
TNM staging	X5	I–II = 0, III–IV = 1
Expression of miR-4739	X6	Hig = 0, low = 1

**Table 5:** Multivariate analysis of prognosis of patients with GC.

Variables	B	S.E	Walds	P	OR	95%CI
Age	0.395	0.206	1.952	0.214	1.506	0.791–2.142
Degree of differentiation	1.191	0.436	4.508	0.038	3.284	1.715–6.822
Lymph node metastasis	1.293	0.511	5.106	0.025	3.704	1.992–5.846
Tumor diameter	1.336	0.694	5.708	0.019	3.809	1.278–7.328
TNM staging	0.741	0.319	5.582	0.021	1.592	1.105–2.086
Expression of miR-4739	0.663	0.312	6.428	0.009	1.905	1.069–3.158

## Discussion

At present, GC is generally confirmed by gastroscopy and biopsy of the lesion. Due to the invasive nature of gastroscopy operation, this examination method cannot be popularized as a routine physical examination item at present, so GC is still not able to achieve early detection and treatment (17, 18). For the treatment of GC, surgery, radiotherapy and chemotherapy are often used. Every GC patient has different responses to the same treatment. If we can timely understand the body characteristics of each patient and implement different treatment plans for different patients, namely personalized treatment, GC treatment will be further optimized (19, 20). In recent 20 years, the incidence and prevalence of GC in China have increased to varying degrees, especially the incidence of GC in young and middle-aged people in recent 10 years. With the continuous development of medical technology, the survival period of GC has been prolonged, but the mortality rate of GC patients is still high (21, 22). Therefore, it is very important to find tumor markers with high sensitivity and specificity for the diagnosis of GC. Studies have shown that miRNA has a specific expression spectrum in a variety of tumor tissues, and its overexpression may promote the growth and proliferation of tumor cells to a certain extent, thus promoting tumor progression (23, 24). Some studies have also pointed out that miRNA may play a role in promoting and inhibiting tumorigenesis (25). Abnormal regulation of miRNA is usually accompanied by epigenetic changes in genes, such as gene deletions, mutations and abnormal changes in DNA methylation level. MicroRNAs are a series of small, non-coding single-stranded RNA fragments in the body, which can regulate the translation and degradation of mRNA by ligating clip pairs in the untranslated region at the 3′ end of mRNAs, thereby affecting the proliferation and differentiation of histiocytes (26, 27). With the deepening of research on microRNAs, more and more research results show that in the process of occurrence and development of many tumors, the expression level of microRNAs is often abnormally increased or decreased (28). As a member of microRNAs, studies have found that Mir-4739 can play an inhibitory role in prostate cancer, and down-regulation of its expression can promote the proliferation. In GC, mir-4739 expression level was up-regulated in GC cells with down-regulated oncogene expression level.

The results of this study showed that the relative expression level of miR-4739 in GC tissues was (0.39 ± 0.06), lower than that in paracancerous tissues (1.18 ± 0.19). These results indicated that the miR-4739 was closely related to the occurrence of GC. The reason was analyzed as follows: overexpression of miR-4739 inhibited the expression of a variety of cell surface molecules, thus inhibiting the migration of tumor cells. The AUC of miR-4739 in the diagnosis of GC was 0.705. When the Youden Index was 0.320 and the optimal cutoff value was 0.37, the sensitivity was 95.30% and specificity was 36.70%. These results indicated that miR-4739 had certain diagnostic value in patients with GC. Further analysis results in this study showed that abnormal expression of miR-4739 was related to tumor differentiation degree, lymph node metastasis, tumor diameter and TNM stage of patients. Patients with low differentiation degree, lymph node metastasis, large tumor diameter and high TNM stage had low expression level of miR-4739. During the follow-up period, 26 of 96 patients died, and the survival rate was 72.92%(26/96). The median survival time was 29 months in the miR-4739 LE group, which was horter than 39 months in the miR-4739 HE group. In addition, univariate and multivariate analyses showed that differentiation, lymph node metastasis, tumor diameter, TNM stage, and miR-4739 expression were all independent factors affecting the prognosis of the patient. Among them, patients with low differentiation, lymph node metastasis, large tumor diameter, high TNM staging and low expression level of miR-4739 have poor prognosis. All these have indicated that miR-4739 level can predict the prognosis of GC patients.

The deficiency of this study is that only the expression of Mir-4739 in GC tissues was detected, and analysis of Mir-4739 may be able to diagnose and predict the prognosis of GC patients. However, the specific mechanism of mir-4739 was not explored in this study, which needs to be further explored in the future.

## Conclusion

The expression of miR-4739 in GC tissue was down-regulated, and its level was related to the degree of differentiation, lymph node metastasis, tumor diameter, and TNM stage. The expression level of miR-4739 has certain diagnostic value for patients with GC, and the prognosis of patients in LE group was worse than that in HE group.

## Data Availability

The original contributions presented in the study are included in the article/supplementary material, further inquiries can be directed to the corresponding author/s.

## References

[1] Song Z Wu Y Yang J Yang D Fang X. Progress in the treatment of advanced gastric cancer. Tumour Biol. (2017) 39:1010428317714626. 10.1177/1010428317714626 28671042

[2] Karimi P Islami F Anandasabapathy S Freedman ND Kamangar F. Gastric cancer: descriptive epidemiology, risk factors, screening, and prevention. Cancer Epidemiol Biomarkers Prev. (2014) 23:700–13. 10.1158/1055-9965.EPI-13-1057 24618998 PMC4019373

[3] Zhao Q Cao L Guan L Bie L Wang S Xie B Immunotherapy for gastric cancer: dilemmas and prospect. Brief Funct Genomics. (2019) 18:107–12. 10.1093/bfgp/ely019 30388190

[4] Wu D Zhang P Ma J Xu J Yang L Xu W Serum biomarker panels for the diagnosis of gastric cancer. Cancer Med. (2019) 8:1576–83. 10.1002/cam4.2055 30873760 PMC6488129

[5] Yoshida K Yamaguchi K Okumura N Tanahashi T Kodera Y. Is conversion therapy possible in stage IV gastric cancer: the proposal of new biological categories of classification. Gastric Cancer. (2016) 19:329–38. 10.1007/s10120-015-0575-z 26643880 PMC4824831

[6] Yang K Lu L Liu H Wang X Gao Y Yang L A comprehensive update on early gastric cancer: defining terms, etiology, and alarming risk factors. Expert Rev Gastroenterol Hepatol. (2021) 15:255–273. 10.1080/17474124.2021.1845140 33121300

[7] Thakur B Devkota M Sharma A Chaudhary M. Evidence based surgical approach to locally advanced gastric cancer. J Nepal Health Res Counc. (2019) 17:133–40. 10.33314/jnhrc.v0i0.2055 31455923

[8] Zheng P Chen L Yuan X Luo Q Liu Y Xie G Exosomal transfer of tumor-associated macrophage-derived miR-21 confers cisplatin resistance in gastric cancer cells. J Exp Clin Cancer Res. (2017) 36:53. 10.1186/s13046-017-0528-y 28407783 PMC5390430

[9] Wang QX Zhu YQ Zhang H Xiao J. Altered MiRNA expression in gastric cancer: a systematic review and meta-analysis. Cell Physiol Biochem. (2015) 35:933–44. 10.1159/000369750 25633747

[10] Xu Q Liu JW Yuan Y. Comprehensive assessment of the association between miRNA polymorphisms and gastric cancer risk. Mutat Res Rev Mutat Res. (2015) 763:148–60. 10.1016/j.mrrev.2014.09.004 25795117

[11] Khayam N Nejad HR Ashrafi F Abolhassani M. Expression profile of miRNA-17-3p and miRNA-17-5p genes in gastric cancer patients with helicobacter pylori infection. J Gastrointest Cancer. (2021) 52:130–37. 10.1007/s12029-019-00319-5 31997281

[12] Wang J Ding Y Wu Y Wang X. Identification of the complex regulatory relationships related to gastric cancer from lncRNA-miRNA-mRNA network. J Cell Biochem. (2020) 121:876–87. 10.1002/jcb.29332 31452262

[13] Zhuang J Wan H Zhang X. Electrochemical detection of miRNA-100 in the sera of gastric cancer patients based on DSN-assisted amplification. Talanta. (2021) 225:121981. 10.1016/j.talanta.2020.121981 33592729

[14] Wang X Chen Q Wang X Li W Yu G Zhu Z ZEB1 activated-VPS9D1-AS1 promotes the tumorigenesis and progression of prostate cancer by sponging miR-4739 to upregulate MEF2D. Biomed Pharmacother. (2020) 122:109557. 10.1016/j.biopha.2019.109557 31918265

[15] Dong L Deng J Sun ZM Pan AP Xiang XJ Zhang L Interference with the β-catenin gene in gastric cancer induces changes to the miRNA expression profile. Tumour Biol. (2015) 36:6973–83. 10.1007/s13277-015-3415-1 25861021

[16] Ajani JA D’Amico TA Almhanna K Bentrem DJ Chao J Das P Gastric cancer, version 3.2016, NCCN clinical practice guidelines in oncology. J Natl Compr Canc Netw. (2016) 14:1286–312. 10.6004/jnccn.2016.0137 27697982

[17] Li Z Liu ZM Xu BH. A meta-analysis of the effect of microRNA-34a on the progression and prognosis of gastric cancer. Eur Rev Med Pharmacol Sci. (2018) 22:8281–87. 10.26355/eurrev_201812_16525 30556868

[18] Kono Y Kanzaki H Iwamuro M Kawano S Kawahara Y Okada H. Reality of gastric cancer in young patients: the importance and difficulty of the early diagnosis, prevention and treatment. Acta Med Okayama. (2020) 74:461–66. 10.18926/AMO/61204 33361865

[19] Yoon H Kim N. Diagnosis and management of high risk group for gastric cancer. Gut Liver. (2015) 9:5–17. 10.5009/gnl14118 25547086 PMC4282848

[20] Ruan Y Li Z Shen Y Li T Zhang H Guo J. Functions of circular RNAs and their potential applications in gastric cancer. Expert Rev Gastroenterol Hepatol. (2020) 14:85–92. 10.1080/17474124.2020.1715211 31922886

[21] Mizukami T Piao Y. Role of nutritional care and general guidance for patients with advanced or metastatic gastric cancer. Future Oncol. (2021) 17:3101–09. 10.2217/fon-2021-0186 34047205

[22] Sanjeevaiah A Cheedella N Hester C Porembka MR. Gastric cancer: recent molecular classification advances, racial disparity, and management implications. J Oncol Pract. (2018) 14:217–24. 10.1200/JOP.17.00025 29641950

[23] He X Zou K. MiRNA-96-5p contributed to the proliferation of gastric cancer cells by targeting FOXO3. J Biochem. (2020) 167:101–108. 10.1093/jb/mvz080 31598681

[24] Ding JN Zang YF Ding YL. MiRNA-146b-5p inhibits the malignant progression of gastric cancer by targeting TRAF6. Eur Rev Med Pharmacol Sci. (2020) 24:8837–44. 10.26355/eurrev_202009_22823 32964972

[25] Hu X Zhang M Miao J Wang X Huang C. miRNA-4317 suppresses human gastric cancer cell proliferation by targeting ZNF322. Cell Biol Int. (2018) 42:923–30. 10.1002/cbin.10870 28880489

[26] Shin VY Chu KM. MiRNA as potential biomarkers and therapeutic targets for gastric cancer. World J Gastroenterol. (2014) 20:10432–439. 10.3748/wjg.v20.i30.10432 25132759 PMC4130850

[27] Jafari N Abediankenari S. MicroRNA-34 dysregulation in gastric cancer and gastric cancer stem cell. Tumour Biol. (2017) 39:1010428317701652. 10.1177/1010428317701652 28468587

[28] Hu ML Xiong SW Zhu SX Xue XX Zhou XD. MicroRNAs in gastric cancer: from bench to bedside. Neoplasma. (2019) 66:176–86. 10.4149/neo_2018_180703N439 30509106

